# The Impact of Work Hours on Depressive Symptoms among Koreans Aged 45 and Over

**DOI:** 10.3390/ijerph18030853

**Published:** 2021-01-20

**Authors:** Juyeong Kim, Eun-Cheol Park

**Affiliations:** 1 Department of Public Health, Sahmyook University, Seoul 01795, Korea; jennajyk394@gmail.com; 2 Institute of Health Services Research, Yonsei University, Seoul 03722, Korea; 3 Department of Preventive Medicine, Yonsei University College of Medicine, Seoul 10711, Korea

**Keywords:** middle-aged and older adults, females, males, unemployment, workforce, depressive symptoms

## Abstract

Background: Given the documented importance of employment for middle-aged and older adults’ mental health, studies of the association between their number of work hours and depressive symptoms are needed. Objectives: To examine the association between the number of work hours and depressive symptoms in Korean aged 45 and over. Methods: We used data from the first wave to fourth wave of the Korea Longitudinal Study of Aging. Using the first wave at baseline, data included 9845 individuals. Depressive symptoms were measured using the 10-item Center for Epidemiological Studies Depression scale. We performed a longitudinal analysis to estimate the prevalence of depressive symptoms by work hours. Results: Both unemployed males and females aged 45–65 years were associated with higher depressive symptoms (β = 0.59, *p* < 0.001; β = 0.32, *p* < 0.001). Females working ≥ 69 h were associated with higher depressive symptoms compared to those working 41–68 h (β = 0.25, *p* = 0.013). Among those both middle-aged and older adults, both males and females unemployed were associated with higher depressive symptoms. Those middle-aged female working ≥69 h were associated with higher depressive symptoms. Conclusions: An increase in depressive symptoms was associated with unemployed males and females working ≥69 h compared to those working 41–68 h. Although this association was found among middle-aged individuals, a decrease in depressive symptoms in both sexes was associated with working 1–40 h. Depressive symptoms should decrease by implementing employment policies and social services to encourage employers to support middle-aged and older adults in the workforce considering their sex and age differences.

## 1. Introduction

With rapidly aging populations in many countries, early retirement from the labor force and limited personal finances have necessitated social policies encouraging employment [1,2]. In South Korea (hereafter Korea), one of the most rapidly aging society among developed countries [3], the employment rate of those aged 60–64 and ≥65 years was 50.8% and 25.0% in 2006, and 58.1% and 25.9% in 2016, respectively [4]. In addition, the employment rate of middle aged Korean is 67.7% in 2017 which is 10% above Organization for Economic Cooperation and Development average [5]. The primary reason for the recent trend in employment and its high rate among aging Korean society is their considerable financial distress if they do not continue to work. Additionally, Korea’s social security and long-term health care insurance systems are relatively immature compared to those in other developed countries [6]. Therefore, the economic participation in both middle-aged (aged 45–64 years) and older adults (aged ≥ 65 years) is expected to remain high or even increase.

Given the high rate of employment among middle-aged and older adults, the association between their working conditions and mental health status, the impact of these conditions, in particular, work hours must be examined. Depression is an important indicator of mental health; in Korea, it occurs more commonly in midlife and old age compared to any other period [7]. Clinical depression and depressive symptoms in turn, have been linked to a higher use of healthcare services, higher spending on health care, and a higher mortality rate [8,9]. The beneficial effect of employment on psychological health has been reported in previous studies. The previous studies found that working longer hours was associated with positive physical and psychological effects [10,11,12]. On the other hand, some studies found that longer working hours are associated with higher depressive symptoms [12,13,14]. The primary reason is that opportunities for active engagement tend to decline in later life and this trend tends to adversely impact overall well-being [15]. Therefore, the association between working hours and depressive symptoms is still unclear. Further, most of these studies were analyzed using samples, which included only those aged 65 and over. Therefore, evidence for the association between work hours and depressive symptoms is still limited, especially for both middle aged and older Korean adults.

In seeking the association between work hours and depressive symptoms, gender differences should be considered. Traditional Confucian values, which stress the patriarchal role of men in Korean society, continue to exert their cultural influence on Koreans. As Korea is a male-oriented society, gender role differences may exist in the association between the number of work hours and depressive symptoms, as males have the responsibility of being breadwinner and females provide overall support for their husband [16]. A previous research has shown that employed women are more likely to have depression than men [17]. Therefore, the burden and effect of work hours on mental health may vary by gender. Additionally, the association by gender should consider age difference as physical condition and other characteristics could be different between middle-aged and older adults [18].

Therefore, we investigated the depressive symptoms of employed and unemployed Koreans across sex, stratified by middle-aged in older men and women. We used the nationally representative data of aging Koreans from the Korean Longitudinal Study of Aging (KLoSA) to determine if the number of work hours was associated with any beneficial effects on depressive symptoms, depending on gender and age. Therefore, we tested two hypotheses: (i) that the influence of the number of work hours on depressive symptoms would vary by gender, and (ii) that the association between males’ and females’ numbers of work hours and depressive symptoms would vary between middle-aged and older adults groups.

## 2. Methods

### 2.1. Data

Data for the study were obtained from the 2006, 2008, 2010, and 2012 waves of the KLoSA, which performed by Korean Labour Institute. KLoSA is a nationally representative study of non-institutionalized South Korean adults aged 45 or over across 15 large administrative areas. Therefore, the survey offered in only Korean language. Among those aged 45 and over, those living in Jeju island or those living in household with no member over the age of 45 were excluded [19]. All data are available via a national public database (http://survey.keis.or.kr/ENLCTGO01N.do?mnucd=cfsaklosa1).

The sampling frame of KLoSA includes enumeration districts (EDs), which were identified by Statistics Korea’s 2005 Census. According to this frame, Apartment EDs and Ordinary housing (non-apartment) EDs were total 261,237, excluding Jeju island for a matter of interviewing convenience. Institutionalized persons were also excluded from first wave. However, the persons excluded from the first wave included in the second wave. An effort was made to produce reliable data even from those cities and provinces with small populations. Then, proportionally EDs were allocated in proportion to the all population of the 15 cities and provinces. Within each city or province, EDs were allocated by area type (urban or rural), in proportion to the population of the areas concerned. For the first wave, 1000 sample EDs were chosen, with the object of acquiring a 10,000 valid interviewee. The first wave of the survey, conducted between July and December 2006, involved 10,254 adults (aged ≥ 45 years) from 6171 households. All participants were interviewed face-to-face using a personal computer-assisted interviewing method. Interviewers read out questions out to respondents from computer screens. Then their responses were entered immediately [20]. The second wave of the survey, conducted between July and November 2008, involved re-interviews with 8688 (84.7%) respondents from the first wave. The third wave of the survey in 2010 used 7920 (80.3%) respondents from the first wave. Finally, in the fourth wave of the survey, 7486 (76.1042%) respondents from the original panel were included. 

### 2.2. Sample

We included only individuals with no missing information. Among 10,254 observations in 2006, missing data accounted for educational level (*n* = 6), smoking status (*n* = 2), grandchild care (*n* = 17), contact with close persons (*n* = 321), Center for Epidemiological Studies Depression (CES-D) scale (*n* = 63). Thus, the final sample at baseline was consisted of 9845 observations. The remaining number of follow-up years and the number of individuals included are as follows. Among 8688 observations in 2008, 8388 observations were included after excluding those with missing information for number of cigarette smoked per day (*n* = 1), grandchild care (*n* = 256), CES-D scale (*n* = 43). Among 7922 observations in 2010, 7684 observations were included after excluding those with missing information data for educational level (*n* = 2), number of chronic diseases (*n* = 1), grandchild care (*n* = 2), contact with close persons (*n* = 195), CES-D scale (*n* = 38). Lastly, observations with missing information for work hours (*n* = 150), and education level (*n* = 2), number of chronic disease (*n* = 4), grandchild care (*n* = 1), contact with close persons (*n* = 167), and CES-D (*n* = 49) were excluded among 7640 observations in 2012. Therefore, 8388 from 2008, 7684 from the 2010, and 7267 from 2012 were included.

### 2.3. Outcome Measures

The 10-item Center for Epidemiological Studies Depression scale (CES-D10) served as the outcome measure. The CES-D10 is a brief screening instrument that assesses depressive symptoms experienced during the most recent week. Of the 10 items, 2 are positively phrased (feel pretty good and generally satisfied) and 8 are negatively phrased (loss of interest, trouble concentrating, feeling depressed, feeling tired or low in energy, feeling afraid, trouble falling asleep, feeling alone, and hard to get going) [21,22]. The responses for each item range from 0 (very rarely or less than once a day) to 3 (almost always or 5–7 days during the past week). The summed scores of the 10 items, with scores reversed for the positively phrased items, comprise the outcome measure. Higher scores indicate greater distress. The alpha coefficient for the CES-D10 was 0.79, which was comparable to the score (0.80) obtained in previous reliability studies [22].

### 2.4. Work Hours

The number of total paid work hours per week including weekdays work, holiday work, and extended work in a week (excluding meals and breaks) was assessed via self-report questionnaires. Work hours per week were classified as unemployed (0 h), <40 h, 41–68 h, and ≥69 h. The standard number of work hours is 40 h per week. However, the current Standard Labor Act allows a maximum of 12 h for extended work and 16 h for holiday work, for a total of 68 h per week, during which time workers may be legally forced to work or allowed to work voluntarily [23]).

### 2.5. Control Variables

We used socio-demographic and health-related variables as covariates to examine the net association of work hours for each gender. The socio-demographic variables included age, marital status (married, single/widowed/divorced/separated), educational level (less than elementary school graduate, middle school graduate, high school graduate, and college graduate), equivalent household income level (Q1, Q2, Q3, and Q4), and wealth (Q1, Q2, Q3, and Q4). Age was categorized into middle-aged (45–64 years old) and older adults (≥65 years old). Equivalent household income was calculated as the total household income divided by the square root of the number of household members; these scores were then divided into quartiles: Quartile 1 (Q1: lowest), Q2, Q3, and Q4 (highest). Net wealth represents the value of public and private pensions and total non-labor income: Quartile 1 (Q1: lowest), Q2, Q3, and Q4 (highest).

For the health-related variables, the number of cigarette smoked per day (non-smoker/1–20/≥21), alcohol consumption (yes/no), the number of chronic diseases (0, 1, and ≥2), instrumental activities of daily living (IADL) disability, and cognitive function were measured. The number of chronic diseases was based on self-reports of having been diagnosed by a physician with one or more of the following conditions: hypertension, diabetes, cancer, lung disease, heart problems, stroke, arthritis, and gastrointestinal problems [24]. Functional status was measured using the 10-item Korean Instrumental Activities of Daily Living Scale, which includes items about personal grooming, excursions of short distances, transportation use, making and receiving phone calls, managing money, performing household chores, preparing meals, shopping, taking medications, and doing laundry. If the respondents were dependent on others for one or more IADL, they were divided into two categories, deepening interaction on whether they had a disability to perform IADLs or not: yes (having a disability) or no (having no disability). The Korean version of the Mini Mental State Examination (MMSE) was used to measure cognitive function [25]. The instrument’s total score ranges from 0 to 30; its validity was confirmed in a previous study

Other covariates included living with children (yes or no), employment status (employed, unemployed, retired, out of the labor force), financial support from children (yes or no), contact with close persons (no close persons, once a month or less, three times a week or less; four times a week or more), and grandchild care (yes, no, other).

### 2.6. Statistical Analysis

We calculated the distribution of the participants’ general characteristics at baseline. T-tests and analysis of variance (ANOVA) were used to analyze the mean CES-D10 scores according to the participants’ general characteristics by sex. We ran a generalized estimation equation (GEE), which is a flexible approach to analyzing correlated data from the same individuals over time [26,27]. The outcome variable in our model was depressive symptoms as the continuous variable. To determine whether the probability of the number of depressive symptoms changed over time, we included time (year) in the model as a categorical covariate. The covariates of interest of all participants were added to the model to determine their effects on the probability of reporting any changes in depressive symptoms and to determine the probability of altered depressive symptoms and independent variables, annually. We found the differences between working hours and depressive symptoms according to sex by interaction test (*p* < 0.001). Therefore, we conducted GEE analysis by sex. For all analyses, a *p*-value of 0.05 or lower was considered statistically significant. All analyses were conducted using SAS Version 9.4 (SAS Institute, Inc., Cary, NC, USA).

## 3. Results

Table 1 summarizes participants’ sex-stratified characteristics at baseline for the covariates. Among male, those unemployed comprised 43.7% of the respondents, those working 41–68 h comprised 36.8%, and those working ≥69 h comprised 9.4%. Among female, those unemployed comprised 75.9% of the respondents, 12.9% for those working 41–68 h, and 4.94% for those working ≥69 h. The mean CES-D10 scores were highest among both unemployed males (3.0) and females (3.4), and the lowest among both males (1.9) and females (2.4) working 41–68 h.

Table 2 shows the adjusted effect of work hours on depressive symptoms. Unemployed males were more likely to be depressed than those working 41–68 h (β = 0.42, *p* < 0.001). Female were more likely depressed than male (β = 0.04, *p* < 0.001).

Table 3 shows the adjusted effect of work hours on depressive symptoms by sex. Unemployed males were more likely to be depressed than those working 41–68 h (β = 0.59, *p* < 0.001), whereas females working ≥69 h were more likely to be depressed compared to those working 41–68 h (β = 0.32, *p* < 0.001).

Table 4 summarizes the effect of work hours on participants’ CES-D10 scores, stratified by sex and age group (45–65 years and ≥65 years). Both unemployed males and females aged 45–64 years were more likely to be depressed compared to those working 41–68 h (β = 0.81, *p* < 0.001; β = 0.35, *p* < 0.001). Among those aged ≥65 years, the males working 69 h ≤ were less likely to be depressed compared to 41–68 h (β = −0.26, *p* = 0.034), and the unemployed males and female were more likely to be depressed compared to those working 41–68 h (β = 0.93, *p* = 0.017; β = 0.34, *p* = 0.001).

## 4. Discussion

We investigated the association between work hours and depressive symptoms among Korean aged 45 years and over by sex and age, which has received little attention as a research topic thus far. In a previous study, decreased depression was reported as work hours increased among medical consultants who were mostly middle-aged [28]. Previously, occupational status was most often the variable of interest in studies of elderly employment; therefore, little is known about the association between work hours and depressive symptoms. Several studies have also examined work hours and psychological health. However, work hours were simply divided into part time or full time, which made it impossible to measure the impact of specific ranges of work hours, such as overtime and unemployment (0 h).

In this study, higher depressive symptoms were strongly associated with those unemployed males and the females who worked ≥69 h. Among both males and females, the higher depressive symptoms were associated with unemployed adults among both middle-aged and older adults, but lower depressive symptoms among those males working 1–40 h in both. Middle-aged females who worked ≥69 h showed higher depressive symptoms and older females who worked 1–40 h showed lower depressive symptoms.

This study’s findings can be explained as follows. First, sex differences were found in the association between work hours and depressive symptoms in this sample of older Koreans. Traditional gender roles and patriarchal social norms based on Confucian values may influence the differences in the association stratified by sex. As mentioned, expected gender roles and paternalism of Korean males who usually play the role of breadwinner in a household, may experience the loss of a work role, such as job loss and retirement, as more detrimental to their mental health compared to females [29], The fixed gender roles in the labor market in societies like Korea, where older females provide physical and emotional support for their husbands [16], could lead to differences in the economic and psychosocial importance of employment [30]. Therefore, if older males feel that they do not fulfill the breadwinner role, the emotional burden can have a harmful effect on their mental health [31].

We also found that females are more likely associated with higher depressive symptoms than males. We found that those with lower equivalent household income is associated with higher depressive symptoms than the highest equivalent household income and the effect of low equivalent household income on depressive symptoms was stronger among females. Previously, females were found to be more likely depressed than males [32]. Females are more exposed to social and economic disadvantages during their life [33], and biological differences such as inflammatory, neurotrophic factors between gender could increase females’ depressive symptoms worse than males [34].

This study found an association between females who worked ≥69 h and higher depressive symptoms. A previous study conducted in New Zealand reported that females were less connected with employment and their salary was considered secondary income by their families [35]. Moreover, aged females in Korea usually feel a duty to care for their family, including their husband, adult children, and grandchildren [24,36]. Given the cultural context of Korea, females working ≥69 h might feel that it is their responsibility to provide care even after working long hours. According to the role strain theory, individuals who manage multiple roles experience worse health compared to their counterparts [37,38]. It is also possible that spending long hours in the workplace leaves little time for social and leisure activities, which is considered to be a detriment to the mental health of older adults [39,40].

Second, an association between unemployed middle-aged males and higher depressive symptoms was found. As mentioned, the role as breadwinner for Korean males, particularly in midlife, as per Confucianism and patriarchal social norms; potentially leads to increased depressive symptoms among unemployed middle-aged males. In contrast, an increase in depressive symptoms was associated with middle-aged females who worked ≥69 h. The association between a demanding workload and an increase in depressive symptoms has been found in other studies with adult and aging populations [41]. Korean females must often play multiple roles (caring for husbands, housework, and grandchildren in some cases), which is associated with poor health [37]. The physical and psychological burden of managing multiple roles, accompanied by long work hours, might have a negative influence on the mental health of middle-aged females working ≥69 h.

Lower depressive symptoms were associated with both males and females. There are several possible reasons for this finding. In a previous study, part-time work helps for better mental health because of positive effects of part-time work such as social network, financial remuneration and support, work-life balance, which is important to better mental health in the later years [42].

There are some strengths and limitations of this study. A large, nationally representative sample was used; therefore, the results can be generalized to South Korean adults over the age of 45 years. On the other hand, we cannot dismiss the possibility of reverse causation between depressive symptoms and work hours as depression might have affected absenteeism or unemployment hence, work hours. Moreover, possible covariates such as treatment of chronic diseases, seasonal factors, job satisfaction, unpaid work, and part-time work were not considered in this study. Finally, this study examined cross-sectional association between work hours and depressive symptoms with panel data. Although we tried analyzing lag effect of work hours on depressive symptoms, we could not find any statistically significant association. Further research is needed, such as more sophisticated longitudinal analysis methods, to find the effect of work hours on depressive symptoms.

The study’s results have several policy implications. The different results found in the associations between work hours and depressive symptoms by gender and age suggest the need for policies that are age-/sex-specific. Government efforts to encourage this do exist; however, several limitations have been reported. There is a shortage of job opportunities with good-quality working conditions [43]. Even within an elderly employment-support program, the needs of each person must be considered, including desired work hours. Furthermore, government efforts are needed to motivate employers to hire older adults; for example, providing economic incentives, such as subsidies and tax deductions to companies hiring elderly employees [44].

## 5. Conclusions

This study found an association between depressive symptoms and unemployed Korean males and females working ≥69 h compared to those working 41–68 h. Although this association was found among the middle-aged adults, lower scores on the depressive symptoms scale was associated with those working 1–40 h for both sexes. Therefore, age-/sex-specific policies must provide support for employed older adults, especially in Korea, which is experiencing rapid population growth and has no mature social security system for retirees. In the future, longitudinal studies should provide evidence for the impact of work hours or changes in work hours on depressive symptoms.

## Figures and Tables

**Table 1:** General characteristics of the participants by CES-D10.

Variable	Male						Female					
	N	% ^a^	Mean	±	S.D	*p*-Value	N	% ^b^	Mean	±	S.D	*p*-Value
Working hours												
Unemployed (0 h)	1867	43.7	3.0	±	2.6	<0.0001	4226	75.9	3.4	±	2.8	<0.0001
1–40 h	436	10.2	2.1	±	2.1		348	6.3	2.9	±	2.7	
41–68 h	1571	36.8	1.9	±	1.8		721	12.9	2.4	±	2.4	
≥69 h	401	9.4	2.2	±	2.2		275	4.9	2.9	±	2.6	
Education level												
Lower than elementary school graduate	1351	31.6	3.1	±	2.6	<0.0001	3272	58.7	3.8	±	2.9	<0.0001
Middle school graduate	728	17.0	2.5	±	2.4		854	15.3	2.7	±	2.4	
High school graduate	1443	33.8	2.1	±	2.1		1178	21.2	2.2	±	2.2	
Upper than college graduate	753	17.6	1.8	±	1.8		266	4.8	1.8	±	1.8	
Age												
Mean ± SD			61.4	±	10.3				62.1	±	11.5	
Marital status												
Married	285	6.7	2.3	±	2.2	<0.0001	1778	31.9	2.8	±	2.5	<0.0001
Single/widowed/divorced/separated	3990	93.3	4.2	±	3.1		3792	68.1	4.1	±	2.9	
Equivalent Household income												
Q1 (Low)	922	21.6	3.1	±	2.6	<0.0001	1500	26.9	3.9	±	2.9	<0.0001
Q2	1084	25.4	2.7	±	2.5		1482	26.6	3.5	±	2.8	
Q3	1360	31.8	2.3	±	2.2		1601	28.7	2.7	±	2.5	
Q4 (High)	909	21.3	1.7	±	1.8		987	17.7	2.3	±	2.3	
Wealth												
Q1 (Low)	1080	25.3	3.0	±	2.7	<0.0001	1917	34.4	3.9	±	3.0	<0.0001
Q2	970	22.7	2.6	±	2.4		1210	21.7	3.3	±	2.7	
Q3	1112	26.0	2.2	±	2.0		1216	21.8	2.7	±	2.4	
Q4 (High)	1113	26.0	2.0	±	2.0		1227	22.0	2.4	±	2.3	
Number of cigarette smoked per day												
Non-smoker	2589	60.6	2.6	±	2.4	0.020	5402	97.0	3.3	±	2.8	<0.0001
1–20	1533	35.9	2.5	±	2.5		162	2.9	4.7	±	3.2	
≥21	153	3.6	2.2	±	2.3		6	0.1	6.5	±	3.3	
Drinking status												
Yes	1574	36.8	2.7	±	2.5	0.017	4536	81.4	3.2	±	2.7	0.185
No	2701	63.2	2.3	±	2.2		1034	18.6	3.1	±	2.7	
Number of chronic diseases												
None	2466	57.7	2.1	±	2.1	<0.0001	2727	49.0	2.5	±	2.4	<0.0001
1	1166	27.3	2.7	±	2.5		1675	30.1	3.5	±	2.8	
2+	643	15.0	3.4	±	2.8		1168	21.0	4.4	±	3.0	
IADL disability												
Yes	695	16.3	2.9	±	2.6	0.000	700	12.6	4.5	±	3.0	<0.0001
No	3580	83.7	2.3	±	2.3		4870	87.4	3.1	±	2.7	
MMSE												
Mean ± SD	22.6 ± 5.3						23.4 ± 6.5					
Living with children												
Yes	2428	56.8	2.2	±	2.2	0.302	3191	57.3	3.0	±	2.7	0.845
No	1847	43.2	2.7	±	2.5		2379	42.7	3.5	±	2.8	
Financial support from children												
Yes	3137	73.4	2.2	±	2.2	0.004	4025	72.3	3.0	±	2.7	0.095
No	1138	26.6	2.9	±	2.5		1545	27.7	3.7	±	2.8	
Contact with close persons												
No close person	25	0.6	3.7	±	3.6	<0.0001	45	0.8	5.1	±	3.1	<0.0001
Once a month or less	964	22.6	2.9	±	2.6		1250	22.4	3.7	±	2.8	
Three times a week or less	783	18.3	2.4	±	2.3		979	17.6	3.1	±	2.6	
Four times a week or more	2503	58.6	2.2	±	2.2		3296	59.2	3.0	±	2.7	
Grandchild care												
Yes	126	3.0	2.6	±	2.5	<0.0001	420	7.5	3.4	±	2.8	<0.0001
No	2231	52.2	2.7	±	2.5		3286	59.0	3.6	±	2.8	
Other	1918	44.9	2.1	±	2.1		1864	33.5	2.4	±	2.4	
Total (N = 9845)	4275	100.0	2.6	±	2.4		5570	100.0	3.3	±	2.7	

^a^ It presented the distribution of male (*n* = 4275); ^b^ It presented the distribution of female (*n* = 5570).

**Table 2:** Adjusted effect of working hours on CES-D10 score.

Variable	Korean Aged 45 and Over		
	β	S.E	*p*-Value
Working hours			
Unemployed (0 h)	0.42	0.04	<0.0001
1–40 h	−0.03	0.05	0.509
41–68 h	Ref.		
≥69 h	0.07	0.06	0.234
Gender			
Male	Ref.		
Female	0.21	0.04	<0.0001
Education level			
Lower than elementary school graduate	0.59	0.07	<0.0001
Middle school graduate	0.40	0.07	<0.0001
High school graduate	0.17	0.06	0.003
Upper than college graduate	Ref.		
Age	−0.13	0.05	0.007
Marital status			
Married	Ref.		
Single/widowed/divorced/separated	0.54	0.05	<0.0001
Equivalent Household income			
Q1 (Low)	0.40	0.06	<0.0001
Q2	0.34	0.05	<0.0001
Q3	0.15	0.04	0.001
Q4 (High)	Ref.		
Wealth			
Q1 (Low)	0.43	0.05	<0.0001
Q2	0.23	0.05	<0.0001
Q3	−0.06	0.04	0.184
Q4 (High)	Ref.		
Number of cigarette smoked per day			
Non-smoker	Ref.		
1–20	0.15	0.05	0.002
≥21	0.23	0.15	0.125
Drinking status			
Yes	0.17	0.04	<0.0001
No	Ref.		
Number of chronic diseases			
Non-smoker	Ref.		
1	0.41	0.04	<0.0001
2+	0.93	0.05	<0.0001
IADL disability			
Yes	0.68	0.05	<0.0001
No	Ref.		
MMSE	−0.06	0	<0.0001
Living with children			
Yes	0.04	0.06	0.549
No	Ref.		
Financial support from children			
Yes	Ref.		
No	0.03	0.04	0.547
Contact with close persons			
No close person	0.55	0.22	0.014
Once a month or less	0.16	0.06	0.011
Three times a week or less	0.01	0.06	0.975
Four times a week or more	Ref.		
Grandchild care			
Yes	Ref.		
No	0.14	0.16	0.362
Other	0.45	0.17	0.007
Year			
2006	−0.51	0.04	<0.0001
2008	0.25	0.04	<0.0001
2010	0.14	0.04	<0.0001
2012	Ref.		

Ref., reference.

**Table 3:** Adjusted effect of working hours on CES-D10 score by Gender.

Variable	Male			Female		
	β	S.E	*p*-Value	β	S.E	*p*-Value
Working hours						
Unemployed (0 h)	0.59	0.06	<0.0001	0.32	0.06	<0.0001
1–40 h	−0.10	0.07	0.164	−0.01	0.08	0.896
41–68 h	Ref.			Ref.		
≥69h	−0.09	0.08	0.251	0.25	0.10	0.013
Education level						
Lower than elementary school graduate	0.69	0.09	<0.0001	0.62	0.11	<0.0001
Middle school graduate	0.48	0.09	<0.0001	0.43	0.11	0.000
High school graduate	0.27	0.07	0.000	0.13	0.10	0.209
Upper than college graduate	Ref.			Ref.		
Age	−0.17	0.07	0.016	−0.12	0.06	0.050
Marital status						
Married	Ref.			Ref.		
Single/widowed/divorced/separated	1.14	0.11	<0.0001	0.34	0.06	<0.0001
Equivalent Household income						
Q1 (Low)	0.35	0.09	0.000	0.40	0.08	<0.0001
Q2	0.32	0.07	<0.0001	0.32	0.07	<0.0001
Q3	0.22	0.06	0.000	0.05	0.06	0.358
Q4 (High)	Ref.			Ref.		
Wealth						
Q1 (Low)	0.45	0.07	<0.0001	0.42	0.06	<0.0001
Q2	0.21	0.07	0.003	0.24	0.06	<0.0001
Q3	−0.01	0.06	0.879	−0.07	0.06	0.230
Q4 (High)	Ref.			Ref.		
Number of cigarette smoked per day						
Non-smoker	Ref.			Ref.		
1–20	0.11	0.05	0.040	0.50	0.15	0.001
≥21	0.12	0.15	0.454	2.19	0.68	0.001
Drinking status						
Yes	0.28	0.05	<0.0001	0.08	0.06	0.194
No	Ref.			Ref.		
Number of chronic diseases						
None	Ref.			Ref.		
1	0.35	0.06	<0.0001	0.47	0.05	<0.0001
2+	0.81	0.08	<0.0001	1.01	0.07	<0.0001
IADL disability						
Yes	0.61	0.07	<0.0001	0.79	0.08	<0.0001
No	Ref.			Ref.		
MMSE	−0.06	0.00	<0.0001	−0.05	0.00	<0.0001
Living with children						
Yes	0.03	0.10	0.765	0.07	0.08	0.362
No	Ref.			Ref.		
Financial support from children					
Yes	Ref.			Ref.		
No	0.09	0.06	0.158	−0.03	0.06	0.635
Contact with close persons					
No close person	0.42	0.34	0.214	0.48	0.28	0.085
Once a month or less	0.03	0.10	0.726	0.23	0.08	0.003
Three times a week or less	−0.11	0.10	0.265	0.06	0.08	0.432
Four times a week or more	Ref.			Ref.		
Grandchild care						
Yes	Ref.			Ref.		
No	0.19	0.16	0.232	0.11	0.09	0.244
Other	0.40	0.17	0.016	0.08	0.10	0.419
Year						
2006	−0.46	0.06	<0.0001	−0.52	0.05	<0.0001
2008	0.20	0.06	0.001	0.31	0.05	<0.0001
2010	0.14	0.06	0.017	0.14	0.05	0.002
2012	Ref.			Ref.		

Ref., reference.

**Table 4:** Adjusted effect of working hours on CES-D10 score by Age and Gender.

Variable	Male						Female					
	Aged 45–64 Years			65 Years and Older			Aged 45–64 Years			65 Years and Older		
	β ^†^	S.E	*p*-Value	β ^†^	S.E	*p*-Value	β ^†^	S.E	*p*-Value	β ^†^	S.E	*p*-Value
Working hours												
Unemployed (0 h)	0.81	0.11	<0.0001	0.48	0.09	<0.0001	0.35	0.07	<0.0001	0.34	0.11	0.001
1–40 h	−0.11	0.10	0.258	−0.16	0.11	0.125	0.06	0.09	0.561	−0.07	0.13	0.625
41–68 h	Ref.			Ref.			Ref.			Ref.		
≥69 h	0.07	0.09	0.482	−0.26	0.12	0.033	0.22	0.11	0.054	0.28	0.20	0.162

Ref., reference; ^†^ Adjusted for age, marital status, equivalent household income, number of cigarette smoked per day, drinking status, number of chronic diseases, IADL disability, MMSE, living with children, employment status, financial support from children, contact with close persons, grandchild care, and year.
